# Armed to the Teeth—The Oral Mucosa Immunity System and Microbiota

**DOI:** 10.3390/ijms23020882

**Published:** 2022-01-14

**Authors:** Maja Ptasiewicz, Ewelina Grywalska, Paulina Mertowska, Izabela Korona-Głowniak, Agata Poniewierska-Baran, Paulina Niedźwiedzka-Rystwej, Renata Chałas

**Affiliations:** 1 Department of Oral Medicine, Medical University of Lublin, 6 Chodzki Street, 20-093 Lublin, Poland; majaptasiewicz@umlub.pl (M.P.); renata.chalas@umlub.pl (R.C.); 2 Department of Experimental Immunology, Medical University of Lublin, 4a Chodzki Street, 20-093 Lublin, Poland; paulinamertowska@gmail.com; 3 Department of Pharmaceutical Microbiology, Medical University of Lublin, 20-093 Lublin, Poland; iza.glowniak@umlub.pl; 4 Institute of Biology, University of Szczecin, Felczaka 3c, 71-412 Szczecin, Poland; agata.poniewierska-baran@usz.edu.pl

**Keywords:** oral microbiota, oral mucosa, immunity system

## Abstract

The oral cavity is inhabited by a wide spectrum of microbial species, and their colonization is mostly based on commensalism. These microbes are part of the normal oral flora, but there are also opportunistic species that can cause oral and systemic diseases. Although there is a strong exposure to various microorganisms, the oral mucosa reduces the colonization of microorganisms with high rotation and secretion of various types of cytokines and antimicrobial proteins such as defensins. In some circumstances, the imbalance between normal oral flora and pathogenic flora may lead to a change in the ratio of commensalism to parasitism. Healthy oral mucosa has many important functions. Thanks to its integrity, it is impermeable to most microorganisms and constitutes a mechanical barrier against their penetration into tissues. Our study aims to present the role and composition of the oral cavity microbiota as well as defense mechanisms within the oral mucosa which allow for maintaining a balance between such numerous species of microorganisms. We highlight the specific aspects of the oral mucosa protecting barrier and discuss up-to-date information on the immune cell system that ensures microbiota balance. This study presents the latest data on specific tissue stimuli in the regulation of the immune system with particular emphasis on the resistance of the gingival barrier. Despite advances in understanding the mechanisms regulating the balance on the microorganism/host axis, more research is still needed on how the combination of these diverse signals is involved in the regulation of immunity at the oral mucosa barrier.

## 1. Introduction

The oral cavity is inhabited by a wide variety of microorganisms, the vast majority of which belong to the normal microbiota. However, it is also inhabited by other opportunistic microorganisms that are involved in the development of not only oral diseases but also systemic diseases. The microbiota comprises all living microorganisms (by type), including mainly bacteria, archaea, fungi, viruses, and small protists. This forms the microbiome, commonly defined as the collective genomic content of all microbes living in a specific environment, which (in different amounts and proportions) inhabit different parts of the mouth. The oral mucosa is a barrier against the penetration and colonization of microorganisms. It is constantly exposed to many triggers requiring immune control, including a variety of commensal microbiota, allergens, food damage, and chewing [1,2].

Oral mucosa consists of the epithelium, the basal membrane, the lamina propria, and the submucosa; the mucosal epithelium is a barrier that separates the underlying tissues from the environment. The oral epithelium is differentiated into keratinized as well as non-keratinized, and the places subjected to mechanical irritation while chewing have a keratin protective layer. The keratinized oral mucosa epithelium is composed of four layers: stratum basale, stratum spinosum, stratum granulosum, and stratum corneum. In the non-keratinized epithelium, the stratum basale is followed by the stratum filamentosum and the stratum distendum [2]. Although we can find many different microorganisms in the oral cavity, such as *Streptococcus*, *Actinomyces*, *Porphyromonas*, *Tannerella*, *Fusobacterium*, *Prevotella*, *Veillonella*, *Campylobacter*, *Eikenella*, *and Treponema*, it is the oral mucosa that is responsible for limiting their colonization and secreting various types of cytokines [1,2]. Under certain circumstances, the imbalance between the normal flora of the oral cavity and the pathogenic flora can lead to a change in the ratio of commensalism to parasitism, an increase in the number of opportunistic microorganisms, and their invasion into deeper tissues, leading to the development of disease [3]. Pathological changes in the oral and periodontal mucosa result from the invasion of pathogens (e.g., *Porphyromonas gingivalis* or *Fusobacterium nucleatum*) [4]. Their penetration into the connective tissue triggers immune responses that play a key role in the development and progression of disease, especially in immunocompromised patients [5]. A healthy oral mucosa has many important functions. Due to its integrity, it is impermeable to most microorganisms and constitutes a mechanical barrier against their penetration into tissues. Thanks to the epithelium and the exfoliation of the cells of its outer layer, the mucosa is continuously renewed. The newly formed cells ensure the balance within the mucosa and as they exfoliate, microorganisms in the mouth are also removed. The defensive function of the mucosa is also associated with the presence of special cells of the immune system [6].

In this systemic review, based on the available literature, we present the role and composition of the oral cavity microbiota as well as the defense mechanisms within the oral mucosa which allow the maintenance of a balance between such numerous species of microorganisms. Thanks to these mechanisms, under physiological conditions, periodontitis and inflammation of the oral mucosa do not develop. In this review, we highlight the specific aspects of the oral mucosa protecting barrier and discuss up-to-date information on the immune cell system that ensures microbiota balance. In addition, we present the latest data on specific tissue stimuli in the regulation of the immune system with particular emphasis on the resistance of the gingival barrier.

## 2. Differentiation of the Composition of the Oral Microbiota

The oral cavity is one of the most complex ecosystems spilling over into the human body. It ensures frequent communication with the external environment through the consumption of food, thus conditioning the differentiation of the microbiota of other parts of the gastrointestinal tract, including the extremely important intestinal microbiota [4]. The oral cavity takes up a small space of our body, amounting to about 0.22 m^2^ (human skin is about 2 m^2^, and the intestine is 500 m^2^), and has the most diverse ecological niches that exist within the human body [2,7,8]. This is possible due to the presence of a wide spectrum of different types of tissue surfaces present in the area.

Oral microorganisms have at their disposal both the structurally hard tooth enamel and the flexible and elastic oral mucosa (as indicated in the literature, the difference in the modulus of elasticity between these two extreme niches is a difference of almost 1 million) [9]. The environmental conditions inside the oral cavity also favorably influence the microbiological diversity of the oral cavity. In this constantly humid environment with variable pH, depending on the type of food consumed and oral hygiene, anywhere from 10^10^ to 10^12^ bacteria belonging to nearly 700 species may live, and as recent scientific studies indicate, up to nearly 1180 taxa [2]. The differentiation of the composition of the oral microbiota concerns not only the content of nutrients in saliva and gingival fluid, but also the concentration of vascular ions, oxygen partial pressure, saliva flow rate, surface area, and oral hygiene measures used. All chemical, mechanical, and microbiological properties in the oral cavity determine the ability of individual microorganisms to colonize this zone [10,11,12]. A 2016 study by the team of Nicolae’s found links between salivary pH, inorganic phosphorus and calcium, and susceptibility to tooth decay. The presented research results show that in people susceptible to dental caries there is a statistically significant decrease in the pH value and the content of phosphorus and calcium in relation to people resistant to caries [13].

**Figure 1:**
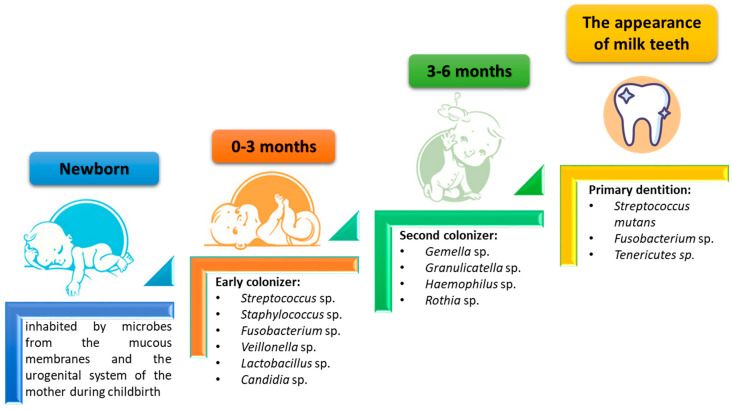
Changes in the composition of the oral microbiota during childhood development (based on [14]).

Therefore, the composition of the oral microbiota is not constant and changes with age. In the early stages of human life, i.e., in newborns and infants, the composition of the oral microbiota is quite poor and its first colonizers are microorganisms from the mother (mainly the genitourinary system and mucous membranes). As shown by research data, the main colonizers of this period (0–3 months) in the oral cavity are gram-positive bacteria such as *Streptococcus* sp., *Staphylococcus* sp., *Fusobacterium* sp., or *Lactobacillus* sp. and the first fungi included is *Candida* sp. These bacteria and fungi prepare the environment for colonization by subsequent groups of microorganisms. Within 3–6 months, colonization with such microorganisms as *Gemella* sp., *Granulicatella* sp., *Haemophilus* sp., or *Rothia* sp. occurs. Subsequent changes in the composition of the oral microbiota occur with the appearance of the first milk teeth, when the consumed food changes, and when oral hygiene increases. Then, the dominant microorganisms in the oral cavity are *Streptococcus mutans*, *Fusobacterium* sp., and *Tenericutes* sp. When the milk dentition is changed to adult dentition, there is an increase in the differentiation of the oral microbiota and the development of a specific set of microorganisms living in the body (Figure 1) [14,15,16].

**Figure 2:**
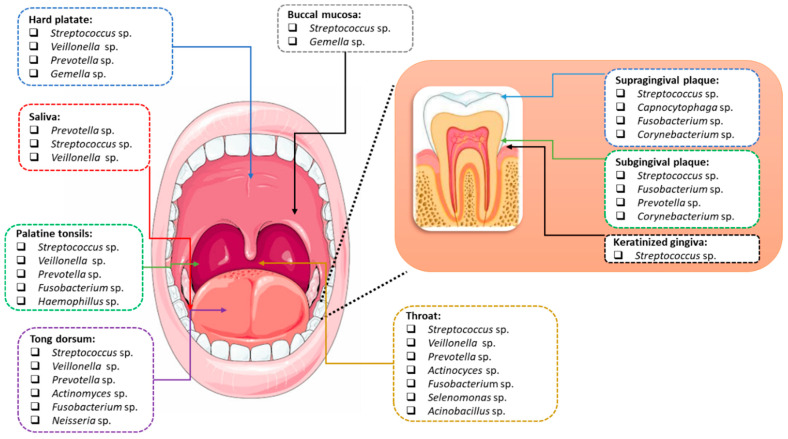
Differentiation of the composition of the oral microbiota depending on the niche (based on [22,23]).

Different areas of the mouth are colonized by different species of microorganisms. The microbiota of the tongue, pharynx, and tonsils vary. Other bacterial cultures can be found on smooth tooth surfaces, in fissures and pits, as well as in carious lesions. In the subgingival region, approximately 400 to 500 microbial populations were detected [17]. The mucosa of the cheeks, the hard and soft palate, and even the vestibule of the mouth are colonized by separate populations of microbiota. Individual species of bacteria mix within the oral cavity and the most common are *Streptococcus*, *Gemella*, *Granulicatella*, *Neisseria*, and *Prevotella* [18]. However, there are areas where specific species of bacteria are detected. The treponemas are usually clustered in the gingival crevice, *Rothia* species usually colonize the tongue or tooth surfaces, *Streptococcus salivarius* mainly colonizes the tongue, and *Simonsiella* colonizes only the hard palate. However, mutans streptococci are, in some cases, not detected in caries lesions (Figure 2) [18,19,20,21].

**Figure 3:**
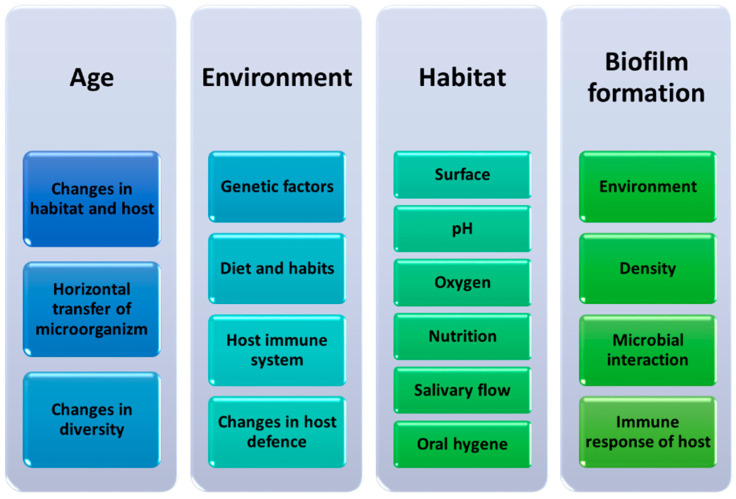
Factors influencing changes in the composition of the oral microbiota during human life (based on [11]).

The composition of the oral microbiota fluctuates throughout human life and takes into account not only age but also diet, genetic, and environmental factors (Figure 2). It should also be noted that each of the ecological niches occurring in the oral cavity may be inhabited by a different group or type of microorganisms. This concerns the differentiation of the surface of these niches and the ability of microorganisms to produce biofilm on their surface (Figure 3) [11].

The purpose of the formation of a specific type of conglomerate on the surfaces of the oral cavity by microorganisms is to protect the bacteria or fungi living inside the multilayer structures against environmental stress, for example, oral hygiene or eating certain types of foods rich in acidic substances, and to provide them with mechanical stability and the ability to communicate through signal transduction, allowing modulation of gene expression and adaptation to changing environmental conditions [24]. The persistence of biofilms formed in the oral cavity is extremely diverse, especially in terms of the place of their formation. Biofilms formed on the surface of the teeth are much more durable than those found on the surfaces of the mucosa. This is largely due to the exfoliation process of the mucosa which significantly reduces the formation of microbial conglomerates. The maturation process of biofilm is also subject to fluctuations due to physical and chemical changes in the oral cavity which is related to the type of food consumed and the degree of hygiene [25]. In addition, significant changes also take place within the biofilm itself. In the originally formed biofilm, the density of such microorganisms is relatively low. As it matures, it is exposed to changing oxygen conditions which not only increases its thickness by increasing the density of the living microorganisms but also the internal layers of conglomerates are cut off from oxygen, creating an anaerobic environment [26,27]. The formation of biofilms on the surfaces of the oral cavity is one of the reasons for the development of oral cavity diseases and systemic diseases as a result of microbiota symbiosis disorders [11,26,27].

## 3. The Importance of Symbiosis (Eubiosis) and Dysbiosis of the Oral Microbiota

Mutual interactions of microorganisms living in the oral cavity are quite varied; here we can distinguish mutualism, commensalism, or parasitism, which are also subject to fluctuations with changing environmental conditions. Under the conditions of the host’s health, the microorganisms of the oral cavity remain in a state of homeostasis with the human body, which in the literature is also called eubiosis [28]. Then, commensal and mutualistic interactions between the living microorganisms that do not affect the health of the host are promoted [28,29]. This does not mean that there is a homogeneous population of microorganisms within the oral cavity, quite the opposite. Thanks to the development of molecular methods, the identification of oral pathogens has become much more accurate and has allowed for the identification of significant differences in the composition of microorganisms. Over the years, culture-dependent techniques leading to the identification of the microbiota in the oral cavity were conducted through broad-range culture/biochemical methods, by which only cultivable and predominant bacteria were reachable, with the risk of missing keystone species in the development of oral diseases. Given the extensive use of molecular biology techniques in recent years, 16S rDNA sequencing has played an important role in the precise identification of isolated bacteria in modern microbiology. Next-generation sequencing (NGS) techniques have revolutionized the study of microbial diversity in recent years. This has allowed for large-scale sequencing projects to be completed in a short time—days, sometimes hours. As a result, scientists not only sequenced the oral microbiome but many clinicians also received an effective tool that allows them to select the appropriate antibacterial treatment and the best method of infection control [30,31].

**Figure 4:**
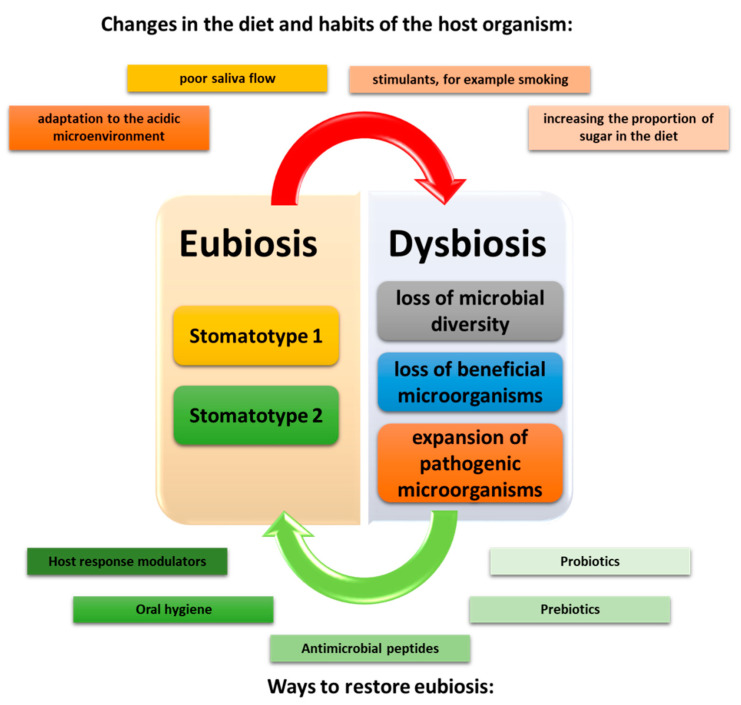
Disorders leading to the formation of dysbiosis of oral microorganisms, taking into account factors contributing to the restoration of eubiosis (based on [26,38]).

Studies in recent years have shown that even during homeostasis there are two types of community distinguished by the microbiota of saliva. They have been referred to by researchers as stomatotypes. Within the first stomatotype, we can distinguish the predominant number of bacteria belonging to Proteobacteria, especially the genus *Neisseria* and *Haemophilus*, while the stomatotype 2 bacteria belong to Bacteroides, especially the genus *Prevotella* and *Veillonella* [28,29,32,33]. Depending on the research, some scientists have also shown the presence of other mixed stomatotypes which include the presence of genera such as *Streptococcus*, *Gamella*, or *Rothia* [28,34]. All changes occurring in the oral cavity resulting from changes in the composition of our diet or environment disturb the state of eubiosis and may lead to the development of the state of dysbiosis in which pathogenic and parasitic interactions between the oral cavity microbiota prevail [11,35,36,37]. The consequence of this condition is the formation of disease states in the host organism. In the literature, we can find three situations that will characterize the state of dysbiosis in the oral cavity. The first is the complete loss of microbial diversity, the second is the loss of beneficial microbes, and the third is the expansion of pathogenic microbes (Figure 4) [26,38].

Loss of the diversity of oral microorganisms is understood as a decrease in the number and genetic variability of living microorganisms, accompanied by a decrease in the species diversity of a given niche. Such a change may lead to disorders of the entire oral cavity ecosystem as a consequence of not only changes in the composition of microorganisms, but also a reduction in the metabolic activity of other bacteria, which may lead to the development of pathogenic bacteria, e.g., cariogenic [27,39,40,41,42]. From the scientific studies conducted so far, it cannot be clearly stated whether the changes in the diversity of the composition of microorganisms between healthy and sick people are the only factor determining the emergence and progression of the host organism disease, especially in the context of periodontal diseases. The research results presented in the literature are contradictory in this field and require further detailed analysis in order to confirm or contradict the hypotheses put forward [43,44,45]. A similar phenomenon concerns the reduction of the number of commensal microorganisms in the oral cavity which were responsible for the protection of the host organism against pathogens and unfavorable metabolites (including carcinogenic compounds) or their participation in the pathways of synthesis and degradation of compounds, e.g., nitrate-nitrite-nitric oxide [46,47,48].

As a result of the loss of microbial diversity and the decline of commensal microorganisms in the oral cavity, pathogenic microbes expand. As indicated in the literature, the growth of pathogenic microorganisms may be correlated with the formation of many disease entities not only located within the oral cavity itself but also systemic diseases. So far, scientists have managed to connect eubiosis disorders of the oral cavity with such diseases as atherosclerosis, Alzheimer’s disease, diabetes, autoimmune diseases, and cancer [26,49,50,51,52]. Studies have shown that the oral microbiome is also involved in wound healing. Due to the fact that this process requires the interaction of many factors, changes and disturbances in the balance in the ecosystem of the oral cavity additionally complicate the healing process [53,54,55,56]. Oral imbalance can adversely affect wound healing, especially in patients with systemic diseases. For example, patients with diabetes mellitus have impaired acute wound healing and this population is prone to developing chronic, difficult-to-heal ulcers. In the oral cavity, saliva acts as a component of innate immunity with antimicrobial activity. In diabetic patients, functional impairment of saliva is observed, both in the composition and flow rate, which impairs wound healing. Furthermore, in other systemic diseases (obesity and leukemia) dysbiosis may lead to disorders of the proper healing process [53,54,55,56]. In addition, oral dysbiosis may lead to premature births and be associated with a higher risk of infection with other microorganisms or viruses, e.g., the current SARS-CoV-2 virus [57,58,59,60,61].

The endogenous human microbial organisms contribute to critical physiological, metabolic, and immune functions, including host differentiation and maturation of the mucosal immune system, detoxification of the environment in the oral cavity, maintaining the barrier of the skin and mucous membranes, regulation of the balance of the immune system, digestion and nutrition of food, energy production, and metabolism regulation. The role of the oral microbiota and its metabolic products in local immune cells training has not been fully explained. Current research indicates both a microbiota-independent and a dependent control of immune homeostasis [62].

## 4. Mechanisms within the Oral Mucosa to Maintain the Microbial Balance

In the oral mucosa, many other cells besides fibroblasts, epithelial cells, and melanocytes exist, such as immune cells. The presence of neutrophils within biofilms was confirmed in different studies. There is little information in the literature on the development of tissue-specific immunity within the gingiva and oral mucosa. The authors focus mainly on the immune system related to the mucosa of the digestive tract, especially the intestines. The oral mucosa is the primary barrier that contains a complex commensal microbiota and is the site where food or airborne antigens are first encountered before entering the gastrointestinal tract. It is important to understand how this barrier effectively regulates immunity and protects the body against the breakdown of defense mechanisms leading to the development of oral or systemic diseases [63,64,65].

It has been shown that commensal microbiota plays a key role in the development and conditioning of local immunity [21,62] with specific microorganisms playing an important role in adjusting the functions of immune cells [66]. Microbiota-independent mechanisms support the establishment of homeostatic immunity. A clear role of commensal colonization in inducing the innate defense of the oral barrier has been demonstrated by some authors [67].

The oral mucosa is the site of first contact for food, airborne antigens, and commensal microorganisms. The response to microbes and antigens is activated at this site and may affect not only local immunity but also subsequent responses at distant sites. The non-keratinizing multilayered squamous epithelium, partially covering the oral mucosa, allows direct contact with environmental antigens. Additionally, the sublingual area is particularly thin and highly vascularized [68].

Shim BS, et al. in 2013 proved on experimental models that the sublingual administration of antigens for vaccination provides effective local and systemic protection. This study indicates the stimulation of systemic immune responses to antigens encountered orally. The induction of specific local immunity in health by oral commensals has not been elucidated in detail. The support for this concept comes from the fact that the characteristics of lymphocytes in healthy oral tissues show the superiority of memory cells [69].

T helper 17 (Th17) cells play an important role as mediators of the immune response. They are involved in immune surveillance and maintenance of the mucosal barrier integrity [70]. The development of Th17 cell-mediated responses in barriers, such as the mucosa, is associated with specific tissue factors, especially colonization by site-specific microorganisms [71,72]. Th17 cells are still poorly understood as cells that supervise specific oral tissues immunity. It has not been definitively established how Th17 lymphocytes are induced in the specific environment of the oral cavity. The essential role of Th17 cells in the protection of the oral immune barrier is particularly apparent in patients with genetic defects in Th17 cell function [73,74]. It has also been proven that Th17 hyperactivity in periodontal tissues promotes inflammatory bone loss and damage to periodontal tissues [75,76]. Understanding the factors involved in the induction and regulation of Th17 cells within the oral mucosa will reveal tissue-specific ways to regulate the immune system in this environment. Therefore, it is important to continue looking for an answer to the question of how Th17 cells are induced and then deregulated in oral inflammation [77].

Dutzan N. et al. (2017) demonstrated the mechanisms controlling the accumulation of Th17 cells in the gingiva. Their research revealed that the gingival interleukin 17 (IL-17)-producing CD4+ T cell population increased with age. Exploring this increase in Th17 cells, they found that the mechanisms controlling CD4+ T cell effector function in the gingiva were different from those operating at other barrier sites. The gingival Th17 cells were dependent upon IL-6-mediated signals. They proved that chewing induces IL-6 and promotes the accumulation of gingival Th17 cells. This mechanism shows that unique principles govern gingival immune homeostasis [77].

It was also found that selected innate antimicrobial defense mechanisms of the oral cavity depend on commensal colonization [21]. Epithelial expression of growth inhibition-specific 6 (GAS6), a ligand of the TYRO3-AXL-MERTK signaling system, has been shown to be commensal-dependent and play a role in controlling inflammation and host-microbiota symbiosis within the oral mucosa [78,79]. The above observations indicate that at a steady state, the oral microbiota locally influences the function of the immune system; it is becoming clear that the immune defense of this barrier is also built independently of commensal colonization.

Immunoglobulins also play a defensive role against microbes in the oral cavity. The main immunoglobulin found in mucosal secretions is the secretory IgA (SIgA) which is the first line of defense in the oral cavity. SIgA controls the oral flora by inhibiting bacterial adhesion to the tooth surface and mucosa [66]. Salivary IgA reactivity to *S. mutans* antigens [11,12] suggests that responses to antigens, especially against major antigens in the *S. mutans* cell surface, such as glucan-binding protein B (GbpB), may affect the ability of *S. mutans* to colonize the oral cavity. This SIgA response to the GbpB antigen may modulate infection by *S. mutans* [80]. On the other hand, it should be mentioned that the interaction between members of oral microbiota mediates prokaryotic resistance to host innate immunity. It was shown that during coculture growth with streptococci, the oral pathogen Aggregatibacter actinomycetemcomitans displays enhanced resistance by sensing the streptococcal metabolite hydrogen peroxide by A. actinomycetemcomitans which stimulates a genetic program resulting in the enhanced expression of the complement resistance protein ApiA. The oxidative stress response regulator OxyR mediates the induction of apiA transcription which is required for coculture resistance to killing by human serum [81].

### Role of Junctional Epithelium Barrier

The non-keratinizing epithelium of the gingival crevice is an exceptionally sensitive part of the oral mucosa barrier. It covers the inner surface of the gums and at the base of the gingival sulcus turns into an incompletely differentiated epithelium, termed junctional epithelium (JE). JE consists of 3–5 layers, and due to its thinness, it is considered a strategic point of the gingival barrier. The connection of JE with the tooth by hemidesmosomes is highly permeable, and the fluid in the gingival crevice (GCF, Gingival Crevicular Fluid) is able to pass continuously. The fluid also plays an essential role in defending against infection, as it contains plasma proteins, defense cells, cytokines, and immunoglobulins [82,83]. The composition of this fluid reflects the local inflammation of the surrounding gums. The JE is constantly exposed to the biofilm and local trauma e.g., while tooth brushing or chewing, which leads to constant immune activation. Through the JE, neutrophils continue to migrate into the oral cavity. Neutrophil detection suggests the existence of microbiota-dependent and independent mechanisms promoting steady-state neutrophil recruitment to the oral barrier [84]. About 95% of all leukocytes that enter the gingival fissure in healthy periodontium are neutrophils [85]. Their number increases during inflammation [86]. It has been estimated that in a human being, within 1 min about 30,000 neutrophils pass through the JE into the gingival crevice [87]. Oral neutrophils show different activation levels and functional states [84,86]. It has not been established whether neutrophils, inside and outside the tissue, mediate microbial control through degranulation, phagocytosis, and secretion of antimicrobial agents [88,89]. Studies in immunodeficient patients suggest additional regulatory roles for tissue neutrophils in addition to microbial destruction. Even in the absence of microbes in the gum tissue, neutrophils are present. The rich immune structure of the gingiva includes neutrophils, constantly migrating through the connecting epithelium, but also lymphocytes—mainly T lymphocytes, some B lymphocytes, and ILCs. Various mononuclear phagocytes are also present at this site [62].

Most studies have focused mainly on T cell populations, especially CD4+ and CD8+, which have been shown to be key mediators in periodontal disease [90,91]. Gingival CD4+ and CD8+ T cell populations produce effector cytokines IFNγ and IL-17 [86,92]. It was found that at the site of the gingival barrier the number of memory T cells increases. In this way, the specific mechanism of an early response to an antigen is enhanced [93]. B cells are present in healthy gingiva, and specific IgA and IgG have been found in oral fluids, but their role in gingival immune homeostasis is not clear [94]. It has been proven that in the course of periodontitis, B lymphocytes play a dual role, both in repair and destruction processes [95,96]. ILC1 and NK are predominant among ILCs, but their ILC functions are still to be determined [86].

**Figure 5:**
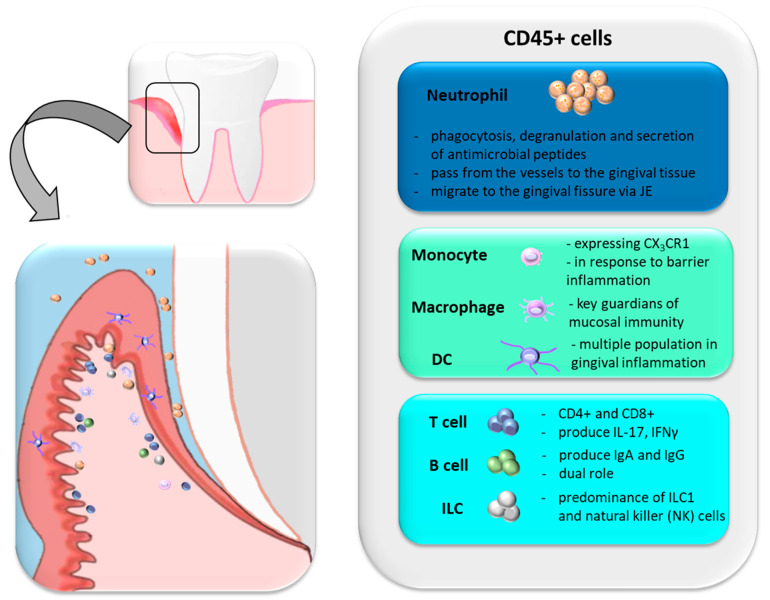
Populations of cells create the gingival barrier and are involved in the response to inflammation (based on [62]).

Mononuclear phagocytes in the gingival barrier are an extensive structure of dendritic cells (DC) [97], monocytes, and macrophages [86]. Monocytes expressing the CX3CR1 + chemokine receptor are accumulated within gingival tissues in bacterial infection [98], indicating that these cells are recruited to damage the gum or oral mucosa barrier. However, the relationship between recruited monocytes and resident macrophages has not been fully explored in the oral cavity area. Although tissue-resident macrophages are key keepers of mucosal resistance in the human body [99], the niche location of macrophages in the gingiva remains to be elucidated. Gingival macrophages are probably involved in the antimicrobial function in the oral cavity but may also be involved in the healing and repair of wounds [100]. Recruited monocytes in the oral cavity can give rise to CD45 + CD11c + CD11b + EpCAM + Langerhans cells (LC) in the gingiva (Figure 5).

## 5. Mechanisms of Autoimmunity Caused by Dysbiotic Changes

**Figure 6:**
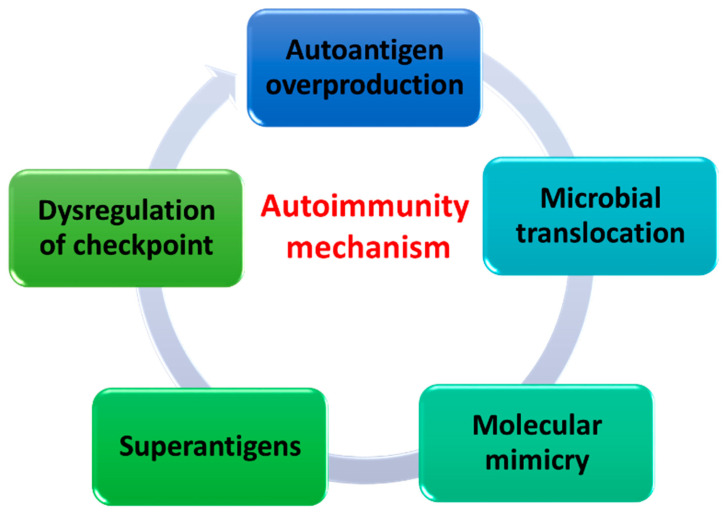
Autoimmune mechanisms caused by changes in oral dysbiosis (based on [101]).

The immune system, which creates a dynamic and complex biological network, is also involved in changing the composition of the oral microbiota throughout the life of every human being. Thanks to the involvement of innate and acquired immunity, every human body learns from an early age to recognize what is present and dangerous to it from what is known to it—developing immunological tolerance. These key relationships contribute to the maintenance of homeostasis and protect the body from getting sick. When immune tolerance disorders occur, the phenomenon of autoimmunity, in which the body attacks its own cells, occurs. Very often, the mechanism of autoimmunity is based not only on genetic and epigenetic differences but also on differences depending on the microorganisms living in the body. There are several autoimmune mechanisms in the literature, which are caused by changes in oral dysbiosis and lead to dysregulation of the proper functioning of the immune system and thus the microbiota-host relationship [101] (Figure 6).

The first mechanism is the overproduction of autoantigens. It occurs when microorganisms begin to produce enzymes responsible for the breakdown of the extracellular matrix, including compounds such as type I collagen, fibrinogen, or fibronectin, which are commonly introduced into the body of every human being and are considered autoantigens [102]. Another way of creating autoantigens is the enzymatic modification of antigens, for example as a result of citrullination, i.e., the conversion of arginine, which is commonly found in peptides, into a rather rare citrulline by peptidylarginine deaminase [101]. This changes the basic properties of the protein such as its charge, conformation, and antigenicity. This results in the formation of antibodies to citrullinated proteins and an alteration of the immune response that may, as indicated in the literature, result in the development of rheumatoid arthritis [103,104].

Another mechanism is microbial translocation, which involves the movement of microorganisms or their metabolic products or cell components via the bloodstream to other organs, altering the local inflammatory response and producing autoantibodies. The defense mechanisms of the human body counteract the excessive multiplication of microorganisms, therefore most of the bacteria found on the nasopharyngeal mucosa (genus such as *Acinetobacter* and *Firmicutes*, as well as *Corynebacterium*, *Propionibacterium*, *Staphylococcus*, *Neisseria*, *Bordetella*, *Streptococcus*, *Pseudomonas*, *Prevotella*, *Haptococcus*, *Pseudomonas*, *Prevotella*, *Porphyromonas*, and *Bacteroides*) do not cause lesions in the equilibrium state of the immune system. However, as a result of invasive procedures, such as intubation or endoscopy, they may be translocated from the oral mucosa and, in changing the ecological niche, contribute to the development of lower respiratory tract infections [105,106,107].

Another mechanism is the application of molecular mimicry by microorganisms. This phenomenon is understood in the literature as a cross-response of the immune system against its own antigens, which is induced by epitopes of microorganisms of similar composition and structure. Among the microorganisms of the oral cavity, we can distinguish a few examples of bacteria that, thanks to molecular mimicry, can induce systemic diseases of their hosts. The first is the protein 60 kDa (Ro60), the autoantigen most commonly found in systemic autoimmune diseases such as Systemic lupus erythematosus (SLE) and Sjögren’s syndrome [108,109]. Studies have shown that it has a 6 amino acid similarity to the *Prevotella denticola* protein containing the VWA domain. Another example would be the small 70 kDa nuclear ribonucleoprotein, which is also an autoantigen involved in SLE, which has a 7 amino acid similarity to *Bacillus cereus* [110]. Oral bacteria can also be involved in the development of diabetes by using molecular mimicry as one way to avoid the immune response. Literature data show that in type I diabetes, *S. mitis* bacteria show a 6 amino acid similarity within the LysM domain to human beta-cell proteins (e.g., GAD65 preproinsulin, islet antigen 2 (IA-2)), which are autoantigens of this disease [111].

The superantigens produced by microorganisms are another mechanism of autoimmunity. Their production results not only in the activation and expansion of a large number of T and B lymphocytes, but also results in the production of significant amounts of regulatory and effector cytokines that modify the host’s defense response. One such example is enterotoxins produced by the ubiquitous staphylococci. Enterotoxin superantigens act in the host organism by stimulating all T lymphocytes carrying reactive Vβ chains. Additionally, they do not require APC processing as is the case with conventional autoantigens. Enterotoxins bind to MHC class II molecules outside the peptide-binding groove and interact with the variable region of the TCR β chain and are involved in the proliferation induction process and effector functions of both CD4 and CD8 T cells [112,113,114].

The last mechanism is the activation or inhibition of immune checkpoints in the human body. The most common points in the literature are inhibitory receptors such as CTL4 (cytotoxic T 4 cell protein) and PD-1 (programmed cell death protein 1), which are key components of the immune system and are involved in the prevention of autoimmunity. The occurrence of chronic infections in the human body leads to the induction of various immunoregulatory mechanisms, such as the production of anti-inflammatory cytokines, activation of regulatory T lymphocytes (Treg), or the expression of immune checkpoints [115]. Research data show that during the development of periodontitis, the expression of CTLA4 on CD4+ T cells increases. For the second checkpoint of PD-1, studies show that there is a link between its expression and susceptibility to autoimmune diseases, including SLE, atopy, and the progression of sclerosis disease [116]. Literature data indicate that PD-1 expression in periodontitis has been assessed to elucidate the presence of immunosuppression, however, no evidence has been found for its role in periodontitis in relation to autoimmunity [117]. In addition, scientists are currently conducting their research into the relationship between possible polymorphisms of genes encoding immunological checkpoints with susceptibility to specific forms of periodontitis in various populations [118,119,120].

## 6. Changes in the Composition of the Oral Microbiota in Diseases

Thanks to the development of molecular techniques based on sequencing (16S rRNA metatranscription, whole-genome sequencing, or metatranscriptomics), scientists were able to successfully determine the diversity of oral cavity-booting microorganisms, even those microorganisms that could not be cultivated under laboratory conditions. This study allowed for the quantitative and qualitative assessment of the composition of the oral microbiota and their changes throughout human life, taking into account the occurrence of disease states. As a result, more and more evidence has emerged that oral microorganisms play a key role in the formation and progression of certain disease entities. Certain species of microbes that colonize the oral cavity have been shown to be associated with oral diseases. Tooth decay, pulpitis, periodontal disease, dry socket, alveolar odor, and odontogenic infections are closely related to the oral microbiota. Some authors have proven that oral microbiota can also be a marker of systemic diseases such as pancreatic cancer [121], type II diabetes [122], pediatric Crohn’s disease [123], heart disease [124], and low birth weight and preterm labor [125]. However, a cause-and-effect relationship has not been definitively proven, and research is ongoing. Table 1 summarizes the most important information relating to the change in oral microbial composition during the development of oral diseases and the development of systemic diseases.

## 7. Conclusions

In recent years the interest in microorganisms colonizing the human body has increased significantly. Increasing attention is paid to the intestinal microbiota and its influence on diseases and general health. Although oral flora is one of the most diverse and its unique human microbiota and impact on health are significant, it still needs further research. It is difficult to determine exactly which organisms a healthy microbiota consists of because they vary from person to person. However, it is known that disruption of homeostasis between microorganisms leads to specific disorders. This imbalance is known as dysbiosis.

The oral mucosa is constantly exposed to numerous environmental factors, but the mechanisms that mediate immune responses are not fully understood. The oral immunological barrier is beginning to be better investigated and recent studies show some similarities with other mucous membranes. However, in the oral environment, the function of certain immune cells remains unknown. Importantly, the anti-inflammatory functions of the oral mucosa’s immune system seem to be crucial for oral mucosa homeostasis. The barrier of the oral mucosa is exposed to unique and diverse microbial communities that have the ability to stimulate the immune system, especially in the case of periodontitis or other inflammatory diseases in the oral cavity.

Despite advances in understanding the mechanisms regulating the balance on the microorganism/host axis within oral mucosa, more research is still needed on how the combination of these diverse signals is involved in the regulation of immunity at this important barrier.

## Figures and Tables

**Table 1:** Associations between the oral microbiota and oral and systemic diseases.

Group of Diseases	Name of Diseases	Associated Organism	References
Oral diseases	Dental caries	Genera: *Neisseria*, *Propionibacterium*, *Selenomonas*, *Lactobacillus*, *Streptococcus*	[40,126]
	Periodontitis	Genera: *Prevotella*, *Fusobacterium*, *Porphyromonas*, *Treponema*, *Aggregatibacter*	[45,127,128,129,130,131,132]
	Oral cancer	Genera: *Prevotella*, *Streptococcus*, *Peptostreptococcus*, *Gemella*	[133,134,135]
Systemic diseases	Pancreatic cancer	Genera: *Porphyromonas*, *Aggregatibacter*, *Leptotrichia*	[136,137]
	Colorectal cancer	Genera: *Lactobacillus*, *Rothia*, *Fusobacterium*	[138,139]
	Cardiovascular diseases	Genera: *Campylobacter*, *Porphyromonas*, *Prevotella*	[140]
	Cystic fibrosis	Genera: *Streptococcus*	[50,141]
	Diabetes	Genera: *Aggregatibacter*, *Neisseria*, *Selenomonas*, *Gemella*, *Fusobacterium*, *Veillonella*, *Streptococcus*	[142]
	Alzheimer’s diseases	Genera: *Porphyromonas*	[143,144]
	Rheumatoid arthritis	Genera: *Veillonella*, *Leptotrichia*, *Prevotella*, *Rothia*, *Lactobacillus*, *Cryptobacterium*	[145,146,147]
